# Reliable, neutral, and comprehensive, or their antithesis? A cross-sectional analysis of electroconvulsive therapy-related video quality across TikTok, BiliBili, and YouTube

**DOI:** 10.3389/fpubh.2026.1796766

**Published:** 2026-04-20

**Authors:** Yuxia Fan, Wenjuan Wang, Hongjiao Xu, Yan Li

**Affiliations:** Qingdao Mental Health Center, Qingdao, Shandong, China

**Keywords:** electroconvulsive therapy, online health information, short-video platforms, social media, video quality assessment

## Abstract

**Background:**

Electroconvulsive therapy (ECT) is a well-established and effective treatment for several psychiatric disorders, however, stigma and misinformation surrounding ECT remain widespread. Social media has become a major source of health information for patients and may influence treatment perceptions and decision-making, yet the quality and reliability of ECT-related content vary substantially across platforms. This study aimed to evaluate the quality, reliability, and dissemination characteristics of ECT-related videos on TikTok, BiliBili, and YouTube, and to identify factors associated with higher informational quality.

**Methods:**

On December 8, 2025, the top 100 videos retrieved using the Chinese keyword “电休克” on TikTok and BiliBili, and the English term “ECT” on YouTube were screened. Videos were independently assessed for attitude toward ECT, content completeness, and overall quality using the Global Quality Scale (GQS), modified DISCERN (mDISCERN), and the Medical Video Evaluation Tool (MQ-VET). Inter-rater reliability was calculated, and non-parametric statistical tests and Spearman correlation analyses were performed.

**Results:**

A total of 71 TikTok videos, 75 BiliBili videos, and 86 YouTube videos were included. YouTube videos demonstrated significantly greater content completeness than those on BiliBili. Overall quality scores were higher on YouTube than on BiliBili, and YouTube also outperformed TikTok in both mDISCERN and total MQ-VET scores. Video uploader identity, presentation format, and content category were differentially associated with video quality across platforms. Engagement metrics were not correlated with video quality on TikTok or BiliBili, whereas positive correlations were observed on YouTube.

**Conclusion:**

Substantial platform-specific differences exist in the dissemination and quality of ECT-related health information. TikTok demonstrates strong user engagement, whereas YouTube provides more comprehensive and reliable content. These findings underscore the importance of platform-tailored, evidence-based strategies to improve the quality and public communication of ECT-related information.

## 1 Introduction

Electroconvulsive Therapy (ECT) is a physical therapy program that induces a wide range of controllable cerebral cortex discharges by releasing a short and appropriate amount of electricity. Modified Electroconvulsive Therapy (MECT) is a modified treatment plan that adds anesthesia induction and muscle relaxant intervention based on ECT (1, 2). Since the birth of ECT therapy in the 19th century, a large number of studies have confirmed its therapeutic effect on various mental illnesses: its effectiveness in treating treatment-resistant depression is 60–80% (3), and the effective rate of treatment for those who do not respond to antidepressants is 50–60% (4, 5), and the reduction rate of patients’ Brief Psychosis Scale (BPRS) score exceeds 40% after 12 treatments, accounting for about 50% (6). ECT has been widely used in the world, and China is the country with the most MECT applications in the world, treating more than 150,000 patients annually (7), indicating that ECT has an irreplaceable position in the non-pharmacological treatment of psychosis.

Despite its established role as a significant intervention for depression, catatonia, schizophrenia, mania, and other psychiatric conditions—and the absence of evidence indicating substantial or permanent neurological damage (8)—ECT remains a subject of persistent controversy. Public perception is predominantly negative, often characterized by a view of ECT as an archaic relic or an instrument of psychiatric maltreatment. This perception is partly attributable to pejorative depictions in social media and cinematic works (9, 10), such as *One Flew Over the Cuckoo’s Nest*, as well as to the transient side effects associated with the procedure, particularly short-term memory impairment. Consequently, patients frequently face considerable distress when considering ECT, leading to ambivalence in decision-making, poor treatment adherence, outright refusal, premature discontinuation of therapy, or the relegation of ECT to a treatment of last resort (8, 11).

Furthermore, the stigma associated with mental illness often drives individuals to seek health information online. Many people with psychiatric conditions avoid formal medical or counseling services due to fears of shaming their families or communities, turning instead to social media for mental health information (12). In 2018, nine out of 10 American adults used the World Wide Web, and 75% of them made medical searches with medical content (13). The rapid expansion of social media has furnished the public with a vast, diverse, and readily accessible repository of content (14). Indeed, compared to textual materials or in-person clinical consultations, video formats are not only more engaging and concise but also reduce the interpersonal demands that may deter some individuals, thereby potentially increasing engagement (15, 16). However, the internet also hosts a substantial volume of inaccurate or misleading health information, which can adversely affect patient decision-making (17, 18). A pertinent example is the case of Yang Yongxin, a Chinese practitioner exposed by television media for using electrical stimulation—wrongly associated with ECT—to treat minors for so-called “internet addiction,” while subjecting them to physical restraint and coercive persuasion. This controversy has repeatedly propelled ECT into negative spotlight on Chinese social platforms, exacerbating public misunderstanding and mistrust of the treatment (19, 20).

TikTok and BiliBili rank among the most influential video platforms in China. While their core user base consists primarily of individuals aged 15 to 35—a demographic characterized by a preference for personalized and high-quality content—the proportion of users over 30 is steadily increasing, reflecting broad societal reach. Meanwhile, YouTube, as the world’s largest video platform, commands a diverse global audience (15, 21). All 3 have become significant channels for public health information. Although platforms such as Kuaishou, Xiaohongshu, Instagram, Facebook, and X (formerly Twitter) also host health-related content, their predominant content formats consist of image-text posts or lifestyle-oriented short videos, which did not align with the specific focus of this investigation on medical knowledge videos. The reach of health science videos and the level of public engagement they garner substantially shape which information disseminates to a broader audience. This phenomenon bears a conceptual resemblance to the evolution from traditional bibliometrics toward altimetric (22), which shifts focus away from conventional citation frequencies toward social media engagement indicators—such as likes, comments, shares, and saves—that, to a considerable extent, serve as a proxy for public attention and societal influence. However, due to variability in uploader expertise and differences in platform oversight, the quality of disseminated medical information is inconsistent, at times containing inaccurate or misleading material (14). Furthermore, health literacy is paramount in enabling patients to effectively comprehend and utilize health information. Given that ECT-related videos often contain substantial textual content, it is essential to consider users’ comprehension capacities. In this regard, the National Institutes of Health, the U. S. Department of Health and Human Services, and the American Medical Association recommend that patient education materials be written at a sixth-grade reading level to ensure accessibility and understanding (23). In recent years, researchers have begun examining the quality of ECT-related content on YouTube. For example, Sabra et al. analyzed 250 English-language ECT videos but relied solely on the Global Quality Scale (GQS) and DISCERN tool, focusing on overall quality and reliability without deeply examining content completeness or underlying attitudes toward ECT (24). Subsequently, Kendirkan et al. introduced an evaluation of content comprehensiveness across 10 clinical dimensions—such as definition, procedure, technical parameters, and indications—yet their analysis was limited to 27 Turkish-language videos, resulting in a restricted sample (25). Moreover, these studies did not move beyond the GQS and DISCERN frameworks and lacked tailored assessment of short-form video features and systematic measurement of treatment attitudes. Thus, existing research exhibits notable limitations:

1. *Platform limitation*: Studies are confined to a single platform (YouTube), lacking cross-platform comparisons—especially across Chinese and English linguistic contexts and long- and short-form video ecosystems.
2. *Incomplete evaluation dimensions*: They fail to systematically assess video stance and lack comprehensive quality assessment tools designed for short-form video formats.
3. *Limited sample representativeness*: Some studies rely on small or single-language samples, raising questions about the generalizability of their conclusions.

This study conducts a cross-platform, cross-sectional content analysis of ECT-related videos on YouTube, BiliBili, and TikTok. Utilizing multidimensional evaluation tools, it explores the relationships among uploader identity, video format, content classification, and video quality indicators. The findings aim to provide an empirical basis for the public to identify reliable information, support professional content creation, and contribute to the development of a healthier platform ecosystem.

## 2 Method

### 2.1 Research design and time

This research employs a cross-sectional content analysis methodology. The video data were sourced from three prominent short-video platforms: BiliBili, TikTok, and YouTube. Data collection was conducted by researchers the period from December 8, 2025, to January 8, 2026.

### 2.2 Ethical considerations

The studies involving humans were approved by the Ethics committee of Qingdao Mental Health Center (QDJWZXWZLL2025046). We confirm that we have read and complied with the Terms of Service/Community Guidelines of TikTok, BiliBili, and YouTube regarding the use of publicly available user-generated content for research purposes. Specifically, data collection was limited to publicly visible, non-identifiable metadata (e.g., likes, comments) and video content. We did not interact with users, access private information, or breach any platform privacy settings, as the research involved no direct subject contact and analyzed only aggregated, de-identified data from public domains. Consequently, according to the local legislation and institutional requirements, the Ethics Committee granted a waiver for the requirement to obtain individual informed consent. The study protocol, which details our adherence to these platform policies, was submitted to the Ethics Committee and is available upon request. The study was conducted in full accordance with the ethical principles outlined in the Declaration of Helsinki.

### 2.3 Search strategy and data screening

The research activity was conducted on December 8, 2025. Searches were performed using the keyword “电休克” on BiliBili and TikTok, and “ECT” on YouTube. Access to YouTube for data collection was conducted exclusively through organizationally authorized network channels, in full compliance with all applicable local regulations and institutional protocols governing internet use. Given that social media content rarely distinguishes between ECT and MECT, we evaluated videos under the broader conceptual framework of ECT-related interventions. The default sorting algorithm on each platform was applied to capture the videos most likely to be presented to a typical viewer. The analysis was limited to the first 100 videos per platform, as prior studies indicate that exceeding this number does not significantly affect analytical outcomes (26, 27). To mitigate the potential influence of browsing history and algorithmic personalization, the following measures were implemented prior to data collection: all Google and associated accounts were logged out, the browsers were configured to incognito mode, and all cookies were cleared to ensure a neutral and unbiased browsing baseline for the data collection process. Given the dynamic nature of online content, all selected videos were archived for subsequent analysis. Videos were included solely on the basis of direct relevance to ECT. Advertisements, film excerpts, and other content unrelated to the thematic focus were excluded. Duplicate videos—defined as identical content uploaded by different users on the same platform—were removed; however, the same video appearing across multiple platforms was retained. Videos on BiliBili and TikTok containing no Chinese-language information, along with videos on YouTube containing no English-language information, were excluded. This approach was adopted to ensure the collected data remained pertinent to the primary linguistic demographics of each platform, thereby preserving the breadth of the intended audience and reducing potential noise introduced by linguistically inaccessible content. Additionally, videos shorter than 10 s were excluded due to insufficient duration for substantive medical communication. 2 researchers independently performed the initial video content extraction. In cases of disagreement, they engaged in discussion to reach a consensus. If a resolution could not be achieved, a third researcher was consulted to arbitrate and determine the final rating. All three raters hold professional titles at the intermediate level or higher and are directly engaged in patient treatment or management at least three times per week. Prior to the study, each reviewer thoroughly familiarized themselves with the guidelines for the assessment tools. All extracted data were systematically recorded using Microsoft Excel on a macOS Sequoia 15.0 system (MacBook Pro), with data collection conducted via a freshly installed Microsoft Edge browser.

### 2.4 Data content and quality assessment

#### 2.4.1 Video features

The extracted video metadata encompassed video source, upload days, duration (seconds), views, likes, favorites, shares, and comments. It is noteworthy that view counts are not publicly available on TikTok, and YouTube similarly does not disclose metrics for retweets or favorites. Video presentation formats were categorized as follows: independently filmed footage, edited videos with narrations, animation, PPT/lecturing, Q&A, and 2 or more of the aforementioned styles. Video content was classified into the following categories: popular science dissemination, research reports, personal treatment reflections and experiential accounts, technical introductions and procedural demonstrations, and advertisements or news reports. Furthermore, the institutional or professional affiliation of video publishers was distinguished, as presented in Table 1.

**Table 1:** Identifications of the video uploaders.

Classification	Connotation
Healthcare-related professionals/Institutions	Certified medical practitioner (psychiatric/non-psychiatric) Certified medical institutions (hospitals/CDC, etc.) Certified medical background science popularization account
Official media/Popular science institutions	Certified news media organizations Certified non-medical professional science popularization organizations (TED, etc.)
General media/Individual users	Personal account without official authentication Sharers who do not clearly identify the patient Non-medical background business or personal accounts
Patient/Patient Family	We can identify whether the uploader is a patient or a family member by watching the video, such as the uploader describing their ECT-related experience with his/her immediate family.

#### 2.4.2 Video quality assessment

In light of the widespread controversy and stigma surrounding ECT, a five-point Likert scale was utilized to evaluate the overall attitude expressed toward the treatment within each video, where a score of 1 denoted entirely negative and 5 entirely positive. The comprehensiveness of a video fundamentally influences the accuracy of information dissemination and the depth of audience comprehension, contributing to a coherent narrative structure and enhancing the video’s credibility. Patients who possess a comprehensive understanding of disease etiology, pathophysiology, treatment modalities, and preventive measures are better equipped to actively engage in and adhere to therapeutic or preventive protocols (28). With reference to established clinical guidelines and expert consensus (29–31), a 10-item checklist was developed to evaluate the completeness of the included videos. The assessed domains are the definition of electroconvulsive therapy (ECT), treatment protocol and historical evolution, therapeutic mechanisms, procedural details, pre- and post-treatment precautions, treatment course, indications and contraindications, efficacy and limitations, potential complications and side effects, cost considerations, and patient perspectives or experiential accounts. A 5-point Likert scale was employed for evaluation, where a score of 1 denotes “highly incomplete” and 5 indicates “highly comprehensive.” Higher scores reflect greater informational integrity within the video content.

The GQS evaluates the accuracy, clarity, and educational value of video content to ensure publicly disseminated information is reliable. Its 5 items are rated on a scale from 1 (poor quality) to 5 (excellent quality). This scale has demonstrated good inter-rater reliability (Cohen’s *κ*) in prior social media research (32).

A multi-instrument approach was adopted to evaluate video quality and information reliability, integrating the Global Quality Scale (GQS), the modified DISCERN instrument (mDISCERN), and the Medical Quality Video Evaluation Tool (MQ-VET). This methodology aims to capture distinctions between the reliability and overall quality of short-form video content, thereby mitigating bias inherent in reliance on a single assessment tool.

In contrast, the mDISCERN instrument is a streamlined version of the DISCERN tool, focusing specifically on information quality rather than overall production quality. It assesses five dimensions: clarity, relevance, traceability, robustness, and fairness. Each dimension is addressed by 1 question, scored binomially (0 = “No,” 1 = “Yes”), yielding a total score range of 0–5 (33).

The MQ-VET is a specialized instrument designed to assess the quality of online medical video content, integrating evaluations of both production quality and informational reliability to furnish a comprehensive appraisal (34). This tool employs a series of detailed metrics, encompassing visual presentation, audio clarity, scientific rigor, logical coherence, and the practical utility of the content. The MQ-VET comprises 15 items, each rated on a five-point Likert scale ranging from “strongly disagree” (1) to “strongly agree” (5). Total scores span from 15 to 75, with higher aggregate scores reflecting superior overall video quality.

#### 2.4.3 Data analysis

Data analysis was conducted utilizing GraphPad Prism 10.0 and R 4.5.1. Normality was assessed using the Shapiro–Wilk test. As all variables deviated from a normal distribution, continuous variables are presented as the median with interquartile range (IQR). Group comparisons were performed using the Mann–Whitney U test for two independent samples and the Kruskal–Wallis test for multiple groups. Inter-rater agreement was quantified by Cohen’s *κ*. Spearman’s rank-order correlation was employed to evaluate associations between engagement metrics and video quality scores. Correlation coefficients (r) range from −1 to 1, where 0 indicates no correlation, values approaching −1 signify a stronger inverse relationship, and values approaching 1 indicate a stronger positive relationship. A threshold of *p* < 0.05 was adopted for statistical significance. All graphical representations were generated using GraphPad Prism version 10.0.

## 3 Result

### 3.1 Video information

**Figure 1:**
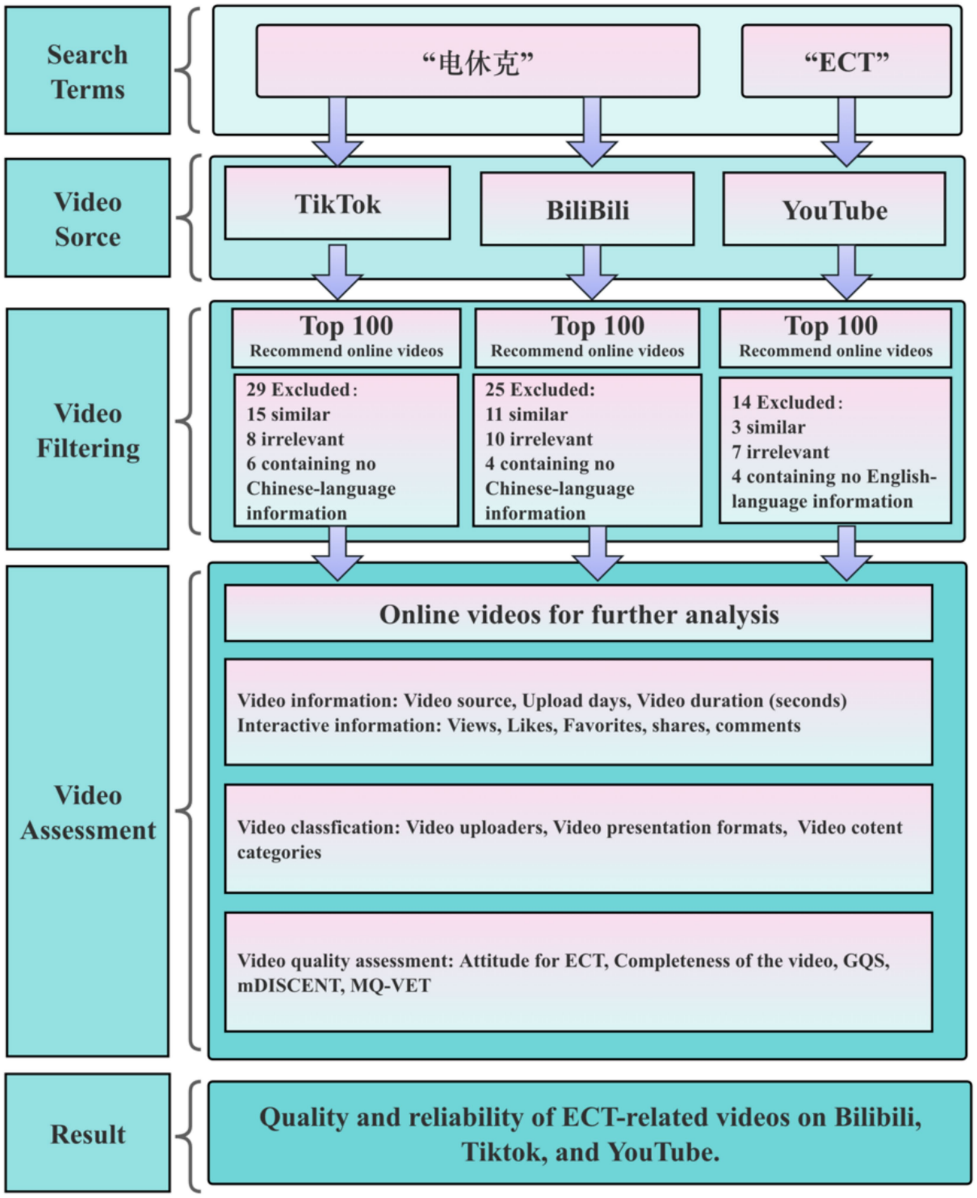
Video selection and exclusion flowchart.

Following the exclusion of duplicate, thematically irrelevant, and linguistically non-conforming videos, this study incorporated 75 videos from BiliBili, 71 from TikTok, and 86 from YouTube (Figure 1). As presented in Table 2, significant differences were observed across the 3 platforms in both upload recency (*H* = 48.142, *p* < 0.001) and video duration (*H* = 19.130, *p* < 0.001). Notably, TikTok videos exhibited the most recent upload times and the shortest durations. In contrast, no significant difference in video duration was detected between BiliBili and YouTube (*U* = 3,177, *p* = 0.871).

**Table 2:** Information of videos about ECT on TikTok/BiliBili/YouTube.

Video information	TikTok (*n* = 71)			BiliBili (*n* = 75)			YouTube (*n* = 86)			*p*-value			
	*M*	Min-Max	IQR	*M*	Min-Max	IQR	*M*	Min-Max	IQR	H_overall_	U_T-B_	U_T-Y_	U_B-Y_
Upload days	455	18–1918	157, 738	1,151	32–2,634	555, 1,532	1074.5	53–6,621	563, 2,398	48.142^***^	1251.5^***^	1258^***^	2732.5
Video duration (seconds)	84	17–3,309	51, 151	205	22–3,489	85.347	227	20–3,948	58, 431	19.130^***^	1651^***^	2004^***^	3,177
Views				3,613	6–315,000	1,300, 7,697	12,134	64–2,444,312	2,603, 44,542				2077.5^***^
Views/30 days				120.43	0.2–10,500	43.33, 356.57	404.47	2.13–81440.4	86.75, 1484.73				2077.5^***^
Likes	528	12–37,000	150, 1,617	114	0–13,000	13,330	136.5	13–330	39.25, 549.75	30.455^***^	1325^***^	1900^***^	2729.5
Likes/30 days	17.6	0.4–1233.3	5, 53.9	3.8	0–433.3	0.43, 11	4.55	0.03–1,300	1.31, 18.33	30.455^***^	1325^***^	1900^***^	2729.5
Collections	123	0–6,718	42, 523	46	0–8,860	12, 105					1608.5^***^		
Collections/30 days	4.1	0–223.93	1.4, 17.4	1.53	0–295.3	0.43, 3.5					1608.5^***^		
Shares	157	2–11,000	38, 695	21	0–1,164	6, 54					1137^***^		
Shares/30 days	5.23	0.07–366.7	1.27,23.17	0.7	0.00–38.80	0.2, 1.8					1137^***^		
Comments	67	0–2,624	16, 220	22	0–1,262	3, 84	21.5	0–1955	2, 92.75	13.521^**^	1836.5^**^	2147.5^**^	3,198
Comments/30 days	2.23	0–87.47	0.53, 7.33	0.73	0–42.07	0.1, 2.8	0.72	0–65.17	0.07, 3.39	13.521^**^	1836.5^**^	2147.5^**^	3,198

H, Kruskal-Wallis; U, Mann–Whitney U; P_T-B_, TikTok VS BiliBili; P_T-Y_, TikTok VS YouTube; P_B-Y_, BiliBili VS YouTube, ^**^p < 0.01; ^***^p < 0.001.

Regarding user engagement, TikTok demonstrated significantly higher counts for likes (*H* = 30.455, *p* < 0.001) and comments (*H* = 13.521, *p* < 0.001), as well as higher corresponding 30-day standardized engagement rates, compared to both other platforms. The collection and share functions are available only on BiliBili and TikTok, with TikTok also significantly outperforming BiliBili in both absolute counts and their 30-day standardized ratios (*U* = 1608.5, 1,137, *p* < 0.001 respectively). Furthermore, YouTube videos attained a significantly higher view count than those on BiliBili (*U* = 2077.5, *p* < 0.001). However, no statistically significant differences were observed between BiliBili and YouTube across several other interaction metrics, including likes (*U* = 2729.5, *p* = 0.093) and comments (*U* = 3,198, *p* = 0.927).

### 3.2 Video classification

**Figure 2:**
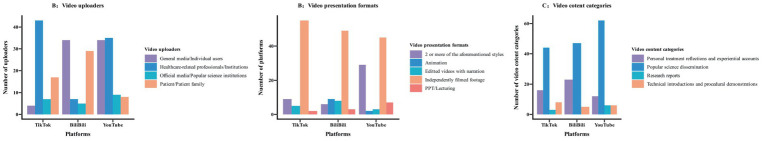
Classification of ECT-related videos on different platforms. **(A)** Illustrate the number of videos uploaded by TikTok, BiliBili, and YouTube. **(B)** Depict the number of video presentation formats of TikTok, BiliBili, and YouTube. **(C)** Present the video content categories of TikTok, BiliBili, and YouTube.

Figure 2 presents the classification of videos across the three platforms (Panels A–C). Regarding video uploaders, TikTok featured the highest proportion of content from healthcare-related professionals or institutions (60.56%). In contrast, BiliBili was dominated by general media or individual users (45.33%) and patients or patient families (38.67%). YouTube content was primarily uploaded by healthcare-related professionals/institutions (40.70%) and general media/individual users (39.53%).

Concerning presentation format, independently produced footage was the most common style on all three platforms (TikTok 77.46%, BiliBili 65.33%, YouTube 52.33%). However, YouTube exhibited a notably higher proportion of videos employing two or more presentation styles (33.72%) compared to TikTok (12.68%) and BiliBili (8.00%).

In terms of content category, popular science dissemination was the dominant classification across all platforms (TikTok 61.97%, BiliBili 62.67%, YouTube 72.09%). This was followed by personal treatment reflections and experiential accounts, which constituted the second most common category on TikTok (22.54%) and BiliBili (30.67%), and the third most common on YouTube (13.95%).

### 3.3 Video quality and reliability

A descriptive analysis was conducted to identify the lowest-scoring items and their score distributions across the 3 platforms (Table 3). As the mDISCERN instrument employs dichotomous scoring (0 = No, 1 = Yes), only the proportion of 0 and 1 responses for each item is presented. The results indicate that, regarding video completeness, all 3 platforms received the lowest scores on the item pertaining to the fee of ECT treatment. For the mDISCERN, all platforms performed most poorly on the item evaluating whether additional sources of information listed for patient reference. Concerning the MQ-VET, the lowest-scoring items on each platform related to the annotation of information timeliness. The assessment of attitude for ECT and the GQS are single-dimensional instruments not designed for item-level analysis and are therefore excluded from this descriptive statistical summary.

**Table 3:** Distribution of the lowest scores in the quality assessment of videos on the 3 platforms.

Video source	Quality assessment	Item	Median (IQR)	Proportion of the highest and lowest scores (*n*, %)	
				Lowest	Highest
TikTok (*n* = 71)	Completeness of the video	Q10: Fees for ECT	1 (1,1)	1:69 (97.18%)	5:2 (2.82%)
	mDISCERN	Q4: Are additional sources of information listed for patient reference?	-	0:60 (84.51%)	1:11 (15.49%)
	MQ-VET	Q1: Dates of updates, if any, are clearly stated	1 (1,1)	1:67 (94.37%)	5:3 (4.23%)
BiliBili (*n* = 75)	Completeness of the video	Q10: Fees for MECT	1 (1,1)	1:66 (88%)	5:2 (2.67%)
	mDISCERN	Q4: Are additional sources of information listed for patient reference?	-	0:66 (88%)	1:9 (12%)
	MQ-VET	Q2: The recording date of the video and date on which the information was accessed are mentioned	1 (1,1)	1:74 (98.67%)	5:1 (1.33%)
YouTube (*n* = 86)	Completeness of the video	Q10: Fees for ECT	1 (1,1)	1:80 (93.02%)	5:1 (1.16%)
	mDISCERN	Q4: Are additional sources of information listed for patient reference?	-	0:60 (69.77%)	1:26 (30.23%)
	MQ-VET	Q2: The recording date of the video and date on which the information was accessed are mentioned	1 (1,1)	1:75 (87.21%)	5:4 (4.65%)

GQS, Global Quality Scale; mDISCERN, modified DESCRN scale; MQ-VET, Medical Quality Video Evaluation Tool.

**Figure 3:**
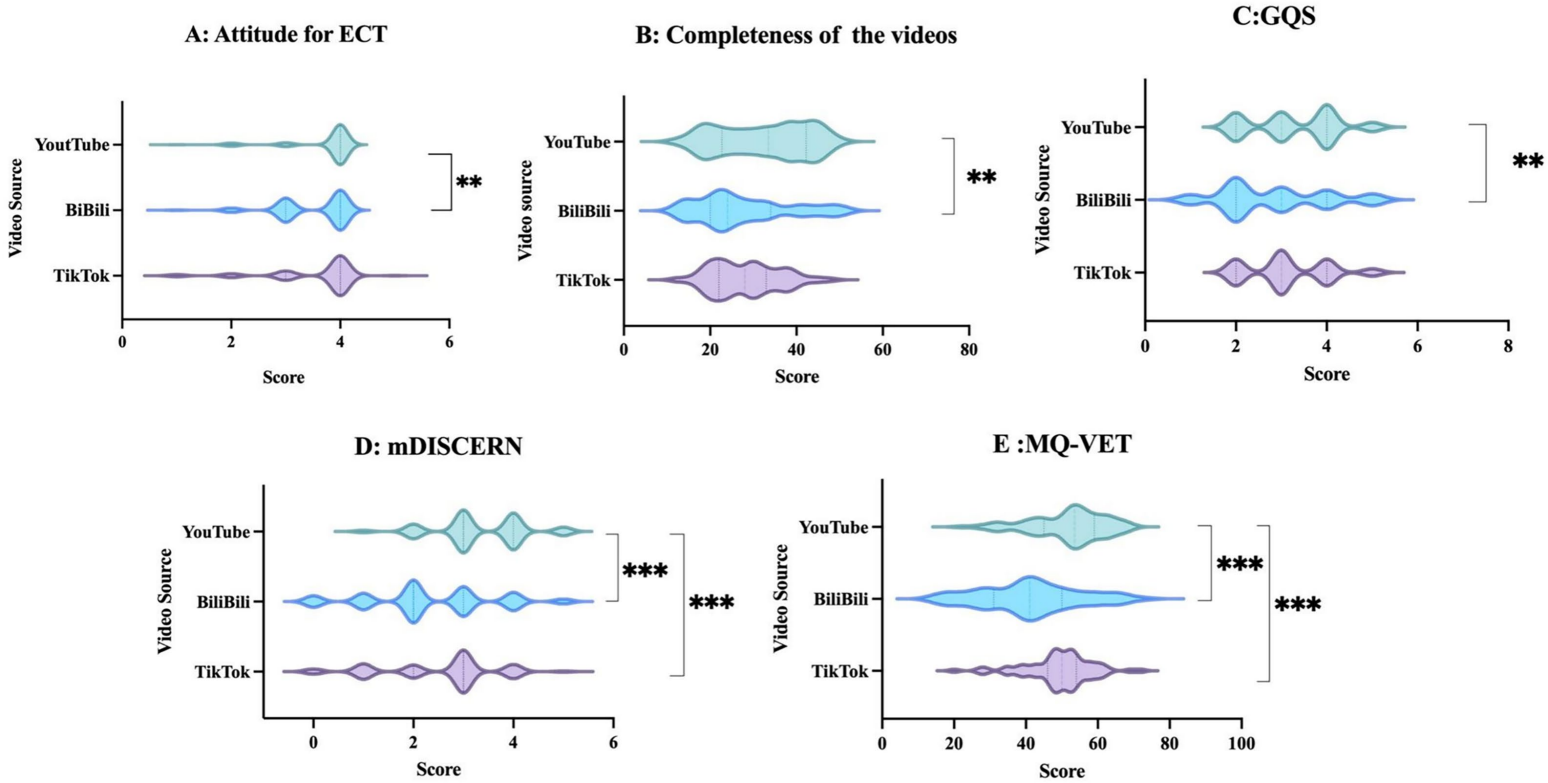
Differences in the quality of ECT-related videos on TikTok, BiliBili, and YouTube **(A–E)**. ^**^indicates *p* < 0.01, ^***^indicates *p* < 0.001. GQS, Global Quality Scale; mDISCERN, modified DESCRN scale; MQ-VET, Medical Quality Video Evaluation Tool.

The inter-rater reliability analysis yielded Cohen’s *κ* coefficients of 0.865 for ECT attitude, 0.964 for video completeness, 0.929 for GQS, 0.932 for mDISCERN, and 0.938 for MQ-VET scores, indicating a high level of agreement between the evaluators across all measured dimensions. The Kruskal-Wallis test revealed significant differences across platforms in attitudes toward ECT scores (*H* = 10.774, *p* < 0.01), video completeness scores (*H* = 9.982, *p* < 0.05), GQS scores (*H* = 31.806, *p* < 0.001), mDISCERN scores (*H* = 10.376, *p* < 0.01), and MQ-VET scores (*H* = 30.787, *p* < 0.001). Figures 3A–E illustrates the cross-platform comparisons for the 5 assessed dimensions—attitude for ECT, video completeness, GQS, mDISCERN, and MQ-VET—along with their composite scores post-Bonferroni correction. Regarding attitudes for ECT, YouTube videos attained a median (IQR) score of 4 (4, 4), which was significantly higher than the scores for BiliBili at 4 (3, 4) (*p* < 0.05). For video completeness, YouTube achieved a median (IQR) score of 33.5 (22.75, 42.25), significantly exceeding the scores for BiliBili at 24 (20, 34) (*p* < 0.01 for both). The GQS median (IQR) scores were 3 (2, 4) across all three platforms. However, YouTube demonstrated significantly higher GQS scores than BiliBili (*p* < 0.01). On the mDISCERN scores, YouTube earned a median (IQR) score of 3 (3, 4), significantly surpassing TikTok’s score of 3 (2, 3) and BiliBili’s score of 2 (2, 3) (*p* < 0.001 for both comparisons). Panel E presents the total MQ-VET scores. YouTube’s median (IQR) score of 59 (53, 64) was significantly higher than both TikTok’s score of 50 (46, 54) (*p* < 0.001) and BiliBili’s score of 41 (31, 50) (*p* < 0.001).

### 3.4 Cross-category differences in video quality scores across different video platforms

**Figure 4:**
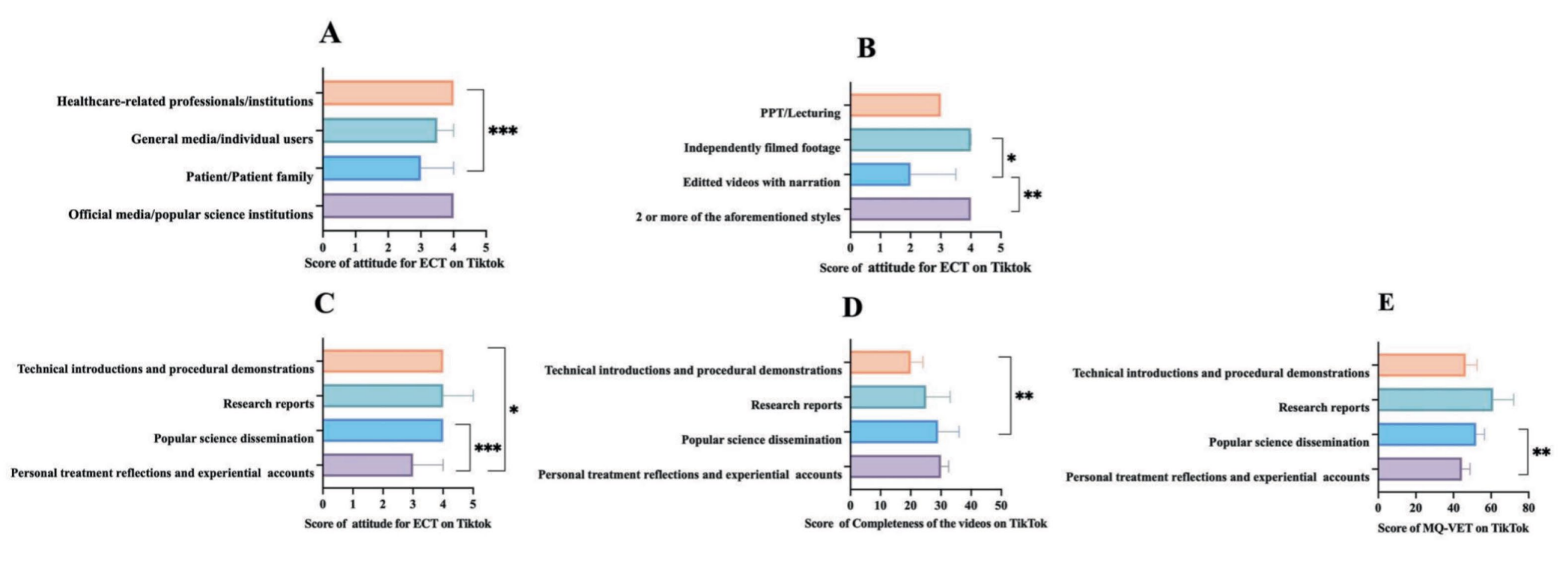
Video quality scores by different video categories on TikTok. **(A)** Variation in scores of attitude for ECT on TikTok, stratified by video uploaders. **(B)** Variation in scores of attitude for ECT on TikTok, stratified by video presentation formats. **(C–E)** Variation in scores of attitude for ECT, completeness of the video scores, and MQ-VET scores on TikTok, stratified by video content. *indicates *p* < 0.05; ^**^indicates *p* < 0.01; ^***^indicates *p* < 0.001. GQS, Global Quality Scale; mDISCERN, modified DESCRN scale; MQ-VET, Medical Quality Video Evaluation Tool.

**Figure 5:**
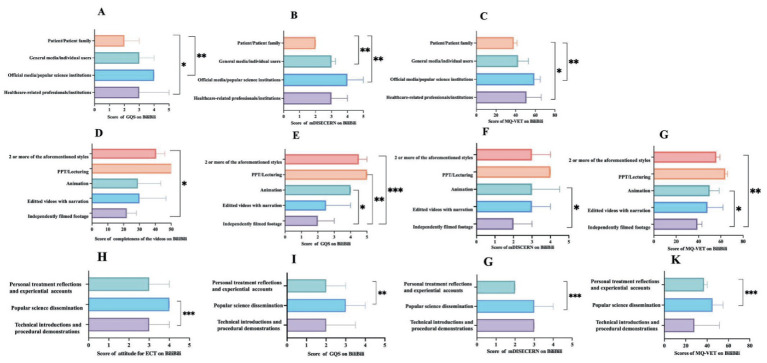
Video quality scores by different video categories on BiliBili. **(A–C)** Variation in scores of GQS, mDISCERN, MQ-VET on BiliBili, stratified by video uploaders. **(D–G)** Variation in scores of video completeness, GQS, mDISCERN and MQ-VET on BiliBili stratified by video presentation formats. **(H–K)** Variation in scores of attitude for ECT, GQS, mDISCERN and MQ-VET on BiliBili, stratified by video content. ^*^Indicates *p* < 0.05, ^**^indicates *p* < 0.01, ^***^indicates *p* < 0.001. GQS, Global Quality Scale; mDISCERN, modified DESCRN scale; MQ-VET, Medical Quality Video Evaluation Tool.

**Figure 6:**
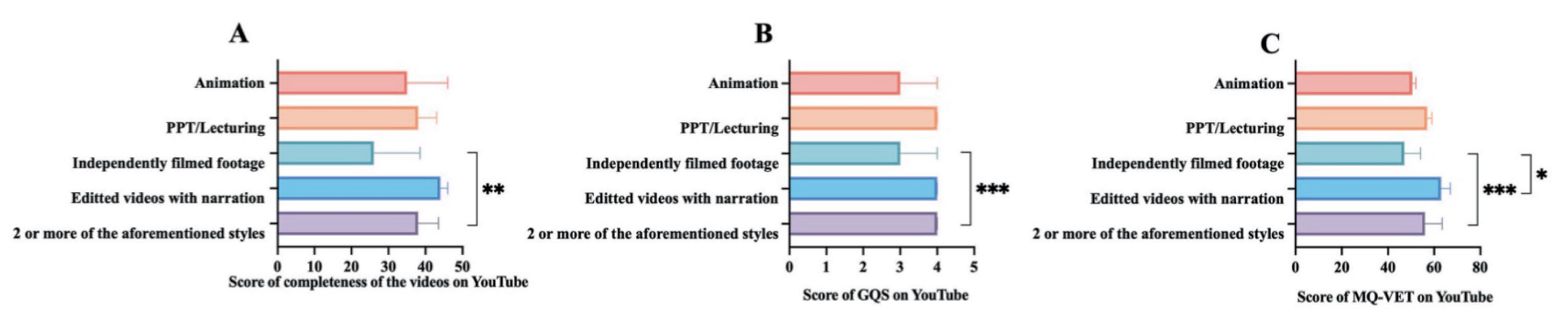
Video quality scores by different video categories on YouTube. **(A–C)** Variation in scores of completeness of the video, GQS scores, and mDISCERN scores on YouTube, stratified by video presentation formats. *Indicates *p* < 0.05, ^**^indicates *p* < 0.01, ^***^indicates *p* < 0.001. GQS, Global Quality Scale; mDISCERN, modified DESCRN scale; MQ-VET, Medical Quality Video Evaluation Tool.

Figures 4–6 illustrate the differentiating characteristics of video quality scores for ECT-related videos across TikTok, BiliBili, and YouTube, stratified by classification. As shown in Figures 4A–E, on TikTok, both video uploaders and presentation formats demonstrated a significant effect on attitude scores (*p* < 0.01 for both). Furthermore, video content significantly influenced multiple quality indicators—excluding the GQS (*p* < 0.05). However, the observed difference in mDISCERN scores across video content did not retain statistical significance following Bonferroni correction. On BiliBili (Figures 5A–J), the variables of video uploaders, presentation formats, and video content significantly affected most quality indicators, with the exception of completeness of the video (*p* < 0.01). Completeness of the video showed a statistically significant difference only across various presentation formats (*p* < 0.01). Notably, post-Bonferroni correction, neither video uploader nor presentation format yielded a significant difference in attitude scores toward ECT. Regarding the YouTube platform (Figures 6A–C), only the video presentation formats exerted a significant effect on the scores for completeness, GQS, mDISCERN, and MQ-VET (*p* < 0.05 for all). Differences in other assessed dimensions were not statistically significant. For comprehensive datasets, please refer to Supplementary Tables 1–3.

### 3.5 Spearman correlation analysis

Spearman correlation analysis revealed distinct associations between engagement metrics and video quality scores across platforms (Table 4): On TikTok, upload days exhibited a negative correlation with completeness of the video (*p* < 0.01). Video duration was positively correlated with both completeness of the video and GQS scores (*p* < 0.001), while the number of shares was negatively correlated with MQ-VET scores (*p* < 0.05). For BiliBili, video duration demonstrated a strong positive correlation with completeness of the video, GQS, mDISCERN, and MQ-VET scores (*p* < 0.001 for all). On YouTube, completeness of the video and GQS scores were positively correlated with all measured interaction indicators (*p* < 0.05 or *p* < 0.01). MQ-VET scores showed positive correlations with all interaction metrics except upload days (*p* < 0.01). Additionally, mDISCERN scores were positively correlated with video duration (*p* < 0.05).

**Table 4:** Spearman correlation analysis between interaction metrics and video quality evaluation scores of different video platforms.

Video source	Variables	Upload days	Duration (seconds)	Likes	Collections	Shares	Comments	Views
TikTok (*n* = 71)	Attitude for ECT	0.014	−0.175	−0.043	−0.038	−0.092	−0.141	-
	Completeness of the videos	−0.238^*^	0.618^**^	−0.122	−0.179	−0.227	−0.051	-
	GQS	−0.111	0.480^**^	−0.079	−0.132	−0.186	−0.022	-
	mDISCERN	−0.197	0.200	0.003	0.034	−0.092	0.039	-
	MQ-VET	−0.158	0.205	−0.174	−0.211	−0.293^*^	−0.177	-
BiliBli (*n* = 75)	Attitude for ECT	0.085	0.158	−0.014	0.07	0.152	0.002	−0.041
	Completeness of the videos	−0.060	0.502^**^	0.014	0.095	0.100	0.011	−0.158
	GQS	−0.036	0.396^**^	−0.022	0.083	0.188	−0.021	−0.039
	mDISCERN	0.011	0.338^**^	0.090	0.159	0.203	0.058	0.016
	MQ-VET	−0.059	0.354^**^	−0.043	0.093	0.188	−0.048	−0.075
YouTube (*n* = 86)	Attitude for ECT	0.025	0.131	0.036	-	-	−0.059	0.108
	Completeness of the videos	0.231^*^	0.692^**^	0.416^**^	-	-	0.422^**^	0.401^**^
	GQS	0.228^*^	0.589^**^	0.442^**^	-	-	0.333^**^	0.447^**^
	mDISCERN	0.050	0.377^**^	0.011	-	-	0.034	−0.030
	MQ-VET	0.067	0.539^**^	0.315^**^	-	-	0.246^*^	0.317^**^

^*^Indicates *p* < 0.05; ^**^indicates *p* < 0.01; ^***^indicates *p* < 0.001.

## 4 Discussion

This study systematically evaluated the characteristics and quality of ECT-related video content on TikTok, BiliBili, and YouTube. TikTok videos exhibited the shortest upload days and durations, yet demonstrated substantially higher interaction rates than those on BiliBili, as well as greater numbers of likes and comments compared with YouTube. This pattern suggests that TikTok may be structurally conducive to fragmented, entertainment-oriented content that prioritizes immediacy and viewer appeal. With regard to content creators, TikTok had the highest proportion of videos produced by healthcare-related professionals or institutions (60.56%), likely attributable to the platform’s stringent verification mechanisms for Chinese medical creators, which preferentially promote content from authenticated accounts (15, 35). In contrast, BiliBili content was predominantly generated by individual users (45.33%) and patients or their family members (38.67%), cultivating an information environment centered on personal experiences and peer support (36). YouTube, by comparison, displayed a more balanced distribution between professional and heterogeneous creator sources. These findings underscore the influential role of platform-specific governance and incentive structures in shaping digital health information ecosystems. Independently filmed footage emerged as the dominant production style across all 3 platforms. However, YouTube featured a significantly higher proportion of videos employing 2 or more presentation formats, a trend likely driven by its longer video durations, a more mature creator economy, and global incentives favoring higher production value. In terms of content categorization, popular science communication constituted the majority (62–72%) of videos on all platforms, indicating that ECT-related discourse remains primarily educational in nature. Notably, the proportion of videos focused on personal treatment reflections and lived experiences was significantly higher on BiliBili and TikTok than on YouTube, highlighting the role of Chinese social media platforms as spaces for patient storytelling and community formation. While such firsthand narratives may help reduce feelings of isolation and offer practical insights, they are inherently limited by subjective perspectives and may not fully reflect comprehensive medical evidence. Consequently, achieving an effective balance between professionally reviewed scientific information and authentic patient experiences represents a critical direction for improving the public communication of electroconvulsive therapy in the future.

The overall findings indicate that YouTube consistently achieves higher video evaluation scores than BiliBili and demonstrates higher scores than TikTok in terms of total mDISCERN and MQ-VET scores. These differences are not only statistically significant but also suggest that the ECT-related content accessed by TikTok and BiliBili users may be limited in terms of completeness, informational depth, informational structure, source reliability, and practical usefulness. Although videos across all 3 platforms generally convey a more favorable attitude toward ECT, a pronounced divergence was observed between YouTube and BiliBili. This disparity may be attributable to the predominance of first-person narratives from patients or their family members on BiliBili, where ambivalence, uncertainty, and adverse treatment experiences—particularly concerns regarding memory impairment—are more openly articulated (24, 25, 37), which may partially contribute to lower overall attitude scores. A similar pattern emerged in video completeness: YouTube content demonstrated an upper–medium level of completeness, whereas videos on TikTok and BiliBili were predominantly at medium or below-medium levels. This difference likely reflects YouTube’s support for and encouragement of long-form content, which affords creators greater temporal scope to systematically address multiple dimensions of ECT, including indications, procedures, benefits, and risks. In contrast, the intrinsic brevity and fragmented narrative structure of short-form video platforms may pose structural constraints on content completeness. This observation underscores the necessity of tailoring ECT science communication to the distinct features of each platform. For short-form platforms like TikTok, an effective strategy could involve developing concise, focused videos, each dedicated to a specific facet of ECT. These could collectively form a sequential or thematic series, allowing users to access a comprehensive understanding through an aggregated set of brief, digestible segments. Notably, information regarding the cost of ECT was the least frequently addressed topic across all platforms. This trend may stem from regional variability in pricing, differences in insurance coverage, and a reluctance among content creators—particularly medical professionals—to oversimplify or risk misinterpretation when discussing cost-related issues in publicly accessible video formats. Although limited evidence suggests that ECT may be associated with relatively high treatment costs (38) and there is economic inequality in access to ECT (39), the influence of economic considerations on patient attitudes and decision-making remains insufficiently explored and warrants further investigation. The absence of such critical information underscores a broader deficiency in supporting users’ digital health literacy, particularly when ECT-related videos on these three platforms serve as key sources of health information. Drawing upon social cognitive theory, external environmental stimuli—such as the perceived reliability of information sources—prompt individuals to recalibrate their internal cognitive processes, which subsequently shape their behavioral responses (40). In this context, the perceived trustworthiness of information sources may function as a mediating mechanism between users’ digital health literacy and their online health information engagement (41). Consequently, the infrequent mention of the financial burden associated with ECT may, on one hand, contribute to informational gaps that potentially delay decision-making and foster treatment hesitation among users. On the other hand, a systemic erosion of user confidence in the reliability of medical information sources may diminish their interaction with and adoption of professional health content, thereby perpetuating an information ecology in which unreliable content proliferates at the expense of credible sources. Regarding mDISCERN scores, the majority of videos across the three platforms failed to disclose their recording or update dates. Given that ECT is a long-established yet continually evolving treatment modality, with ongoing refinements in practices such as anesthesia and muscle relaxation techniques, the omission of update timelines undermines patients’ ability to assess the currency and reliability of the information and conflicts with the evidence-based medicine principle of relying on “the best currently available evidence” (42). Furthermore, analysis of individual MQ-VET items revealed that few videos provided patients with supplementary avenues for obtaining additional information, thereby further diminishing the transparency and traceability of medical content.

We further observed significant variations in video quality scores across different video characteristics. Overall, content produced by medical professionals or institutions, videos employing 2 or more presentation formats, and popular science–oriented videos were associated with more positive attitude scores toward ECT, higher levels of content completeness, and superior overall quality evaluation scores. These findings are consistent with previous research (36, 43) and underscore the importance of creators’ professional backgrounds, science communication–based narratives, and multimodal presentation strategies in enhancing credibility, structural clarity, and informational coherence. However, a persistent challenge is the relatively limited professional contribution of medical professionals and institutions to ECT-related video content, which accounts for only 45.6% of the total across the three platforms. Notably, disparities in video quality scores across video features were more pronounced on BiliBili. This pattern may be explained by the platform’s user composition, which is dominated by general users (45.33%) and patients or their family members (38.67%). On BiliBili, the dissemination of ECT-related knowledge and personal experiences tends to be more informal and everyday in nature, with content often grounded in anecdotal evidence and subjective opinions (44). From a clinical perspective, such life-oriented narratives may lack methodological rigor and comprehensiveness (45) and, in some cases, may reflect bias or skepticism toward ECT. Nevertheless, the treatment experiences conveyed, and the emotional resonance fostered among users remain noteworthy and merit careful consideration, particularly in the context of patient-centered communication. Future research could productively focus on extracting and performing sentiment analysis on user comments associated with ECT-related videos across these platforms. A comparative analysis of such discourse would facilitate a deeper understanding of public attitudes, emotional responses, and prevalent misconceptions regarding ECT. This approach would yield valuable evidence to inform interventions aimed at supporting patient decision-making, mitigating stigma, and addressing widespread concerns.

Furthermore, our analysis revealed no correlation—or even an inverse relationship—between video quality scores and engagement metrics on TikTok and BiliBili. This finding aligns with prior research indicating that high-quality content does not necessarily achieve broad visibility on Chinese short-video platforms (45, 46). This phenomenon may be attributed to the fact that higher-quality videos often present ECT in a more systematic and comprehensive manner. Healthcare-related professionals, mindful of their identity and social responsibility, tend to adopt a rigorous rather than sensationalized approach in titles and thumbnails, which may reduce immediate viewer engagement. Simultaneously, the algorithmic prioritization of videos with high interaction rates on TikTok and BiliBili may further widen the disparity in engagement between professionally oriented content and more emotionally charged or entertainment-focused material (16). In contrast, on YouTube, both the GQS and MQ-VET scores exhibited positive correlations with all interaction metrics and video duration. This likely stems from YouTube’s core algorithm, which optimizes for sustained watch time and meaningful engagement. Moreover, users on the platform often approach content with an information-seeking mindset, valuing depth, accuracy, and credible sourcing. Consequently, thorough, well-structured, and referenced videos are better positioned to meet user expectations and, in turn, generate higher-quality interaction (47, 48).

### 4.1 Implications

This study provides insights for platforms, content producers, patients, and the broader public regarding the overall quality landscape of ECT-related video content. In the context of an information-saturated media environment, misleading and stigmatizing representations of ECT remain widespread (49). Consequently, enhancing patients’ ability to critically evaluate ECT-related information should be a primary priority. Targeted online user education and training programs may improve patients’ health information literacy, thereby enabling more effective screening and appraisal of digital health content. In parallel, video platforms could implement automated health information monitoring systems to identify potential logical flaws—such as extreme data claims or causal fallacies—and embed contextual prompts designed to stimulate users’ critical thinking (50). Equally important is addressing the discrepancy between healthcare professionals’ and patients’ attitudes toward ECT. Attitudes reflect individuals’ predispositions to evaluate people, concepts, or interventions and constitute a key mediating factor linking knowledge acquisition to behavioral decision-making (51). Existing literature indicates a discernible disparity in attitudes toward ECT between healthcare professionals and patients, with negative perceptions frequently reported among patients and their families (52–54), a pattern consistent with the findings of the present study. Such attitudinal divergence may stem from differing perceptions of ECT-related side effects between clinicians and patients, as well as from patients’ limited understanding of ECT, which has been associated with incomplete informed consent (55, 56). Therefore, prior to preoperative ECT consultations, patients should be provided with balanced, comprehensive, and evidence-based information. Exposure to well-structured and impartial educational materials on ECT has been shown to foster more positive attitudes among patients and their families and to increase willingness to consider ECT as a treatment option (57). Accordingly, the implementation of structured and systematic educational interventions represents a critical strategy for improving patient awareness and attitudes toward ECT. Furthermore, an appropriate balance must be achieved between viewer engagement, video length, and the quality of ECT-related content. Central to this balance is a paradigm shift in health communication—from a traditional, one-directional knowledge transmission model to a user-centered cognitive adaptation model. Under the premise of ensuring scientific accuracy, strategic content design should aim to reduce cognitive load and align with users’ attention patterns, thereby facilitating efficient access to and internalization of health knowledge. Finally, established health education assessment instruments such as the GQS, mDISCERN, and MQ-VET have demonstrated broad applicability and reliability. Healthcare professionals can employ these validated tools as effective reference standards to develop more comprehensive and rigorous content guidelines for ECT-related public education materials. This approach would contribute to a systematic elevation in the quality of video-based health communication across platforms.

## 5 Conclusion

This study evaluated ECT-related video content on TikTok, BiliBili, and YouTube, demonstrating that YouTube hosts the highest-quality videos overall, TikTok generates the greatest user engagement, and BiliBili features the largest proportion of content produced by patients or their family members. While YouTube demonstrates a clear superiority in overall video quality metrics, each of the three platforms exhibits distinct areas of relative weakness in their quality assessments. Video quality varied substantially across platforms and was influenced by content creators, presentation formats, and content types, while engagement metrics were not reliable indicators of informational quality on Chinese video platforms. Collectively, these findings highlight the need to bridge attitudinal differences toward ECT among diverse user groups, strengthen users’ health information literacy to improve the identification of potential misinformation, and promote the development of standardized, evidence-based reporting guidelines for ECT-related health education content.

### 5.1 Strengths

This study possesses several notable advantages. First, it employs a cross-platform comparative design that encompasses both short- and long-form video content across diverse cultural contexts, thereby facilitating a more comprehensive assessment of the digital information ecosystem. Second, it utilizes a range of validated evaluation instruments, which enhances methodological rigor and enables a thorough assessment of video content completeness as well as participants’ attitudes toward ECT, thereby improving the generalizability of the findings. Third, by integrating engagement metrics with quality and attitudinal analyses, this study not only elucidates the characteristics of the information itself but also clarifies the relationship between digital visibility and content reliability.

### 5.2 Limitations

This study has several limitations. First, it should be acknowledged that although the video assessment was conducted independently by three researchers, a degree of subjectivity was inherently unavoidable, particularly with regard to the utilization of Likert-scale instruments within the assessment framework. Second, the cross-sectional design precludes the examination of temporal trends and dynamic changes in ECT-related video content over time. Third, platform-specific affordances constrained the collection of certain engagement metrics—such as reposts and favorites on YouTube and view counts on TikTok—which may limit the comparability and interpretability of cross-platform analyses. In addition, although English-language ECT-related videos on YouTube were included, the study may not fully capture the global diversity of linguistic and cultural contexts in which ECT-related information is disseminated. While user comments may offer valuable insights into public perceptions, attitudes, and concerns regarding ECT, they were not systematically analyzed in this study. Future research could incorporate text-based analytic approaches, such as content or sentiment analysis, to examine comment data in greater depth and thereby provide a more comprehensive understanding of public engagement with ECT-related digital content. In addition to video-based social media platforms, patients increasingly seek health information through AI-supported systems and conventional web-based educational materials (58, 59). Studies evaluating the readability of internet-based patient education resources have consistently shown that such materials often exceed recommended readability standards, potentially limiting accessibility (23). Unlike algorithm-driven video platforms that prioritize engagement and emotional salience, traditional web materials are typically more structured but may lack personalization and interactive feedback. AI-supported systems, by contrast, generate context-sensitive responses that may enhance clarity and coherence, yet remain dependent on training data quality and prompt formulation, and concerns regarding factual accuracy and scientific grounding persist (58). These differences highlight that digital health information ecosystems vary not only in format but also in mechanisms of content production, visibility, and quality control, which may ultimately influence how patients construct their understanding of ECT.

## Data Availability

The raw data supporting the conclusions of this article will be made available by the authors, without undue reservation.
