# Supplementary material for: Reliable, neutral, and comprehensive, or their antithesis? A cross-sectional analysis of electroconvulsive therapy-related video quality across TikTok, BiliBili, and YouTube
Source: Front Public Health. 2026 Apr 20;14:1796766. doi: 10.3389/fpubh.2026.1796766 (PMC13136114; doi:10.3389/fpubh.2026.1796766)
Supplement: Supplementary file 1 [file Table_1.docx]

Supplemental Table1. Video Quality Scores by different video categories on TikTok (*n*=71)

| Variables | Attitude for ECT Median（IQR） | Completeness of the videos Median（IQR） | GQS Median（IQR） | mDISCERN Median（IQR） | MQ-VET Median（IQR） |
| --- | --- | --- | --- | --- | --- |
| Video uploaders |  |  |  |  |  |
| Healthcare-related professionals/institutions | 4 (3.5, 4) | 25 (20.5, 33) | 3 (2, 4) | 3 (2, 3) | 51 (47.5, 56) |
| Official media/popular science institutions | 4 (4, 4) | 29 (23, 31) | 4 (4, 4) | 3 (2, 3) | 47 (42, 50) |
| General media/individual users | 3.5 (1.5, 4) | 27.5 (23.5, 31) | 3 (2, 4) | 1.5 (0.5, 3) | 46.5 (33, 55) |
| Patient/Patient family | 3 (2, 4) | 30 (25, 35) | 3 (3, 4) | 3 (2, 3) | 52 (50.5, 54) |
| *P* | 0.001 | 0.373 | 0.595 | 0.605 | 0.123 |
| *H* | 17.26 | 3.125 | 1.89 | 1.846 | 5.780 |
| Video present formats |  |  |  |  |  |
| Independently filmed footage | 4 (3.5, 4) | 29 (21.5, 33.5) | 3 (2.5, 4) | 3 (2, 3) | 50 (46, 54) |
| Editted videos with narration | 2 (2, 3) | 25 (25, 31) | 2 (2, 3) | 1 (1, 2) | 38 (35, 47) |
| PPT/Lecturing | 3 (3, 3) | 35.5 (23, 48) | 3.5 (2, 5) | 2.5 (1, 4) | 45.5 (29,62) |
| 2 or more of the aforementioned styles | 4 (4, 4) | 28 (25, 29) | 3 (3, 4) | 3 (3, 3) | 53 (51, 55) |
| *P* | 0.002 | 0.889 | 0.684 | 0.418 | 0.100 |
| *H* | 14.752 | 0.631 | 1.492 | 2.833 | 6.241 |
| Video cotent |  |  |  |  |  |
| Technical introductions and procedural demonstrations | 4 (4, 4) | 20 (19.5, 23) | 2 (2, 3) | 2 (1, 3.) | 46.5 (44, 52) |
| Popular science dissemination | 4 (4, 4) | 29 (22.5, 36) | 3 (3, 4) | 3 (2.5, 3) | 52 (48.5, 56) |
| Research reports | 4 (3, 4.5) | 25 (18.5, 29) | 2 (2, 3) | 4 (2.5, 4.5) | 61 (49.5, 66.5) |
| Personal treatment reflections and experiential accounts | 3 (2, 4) | 30 (23, 32) | 3 (3, 3.5) | 2 (0.5, 3) | 44.5 (37.5, 48.5) |
| *P* | <0.001 | 0.031 | 0.074 | 0.034 | 0.003 |
| *H* | 18.088 | 8.861 | 6.939 | 8.676 | 13.862 |

Supplemental Table2. Video Quality Scores by different video categories on BiliBili（*n*=75）

| Variables | Attitude for ECT Median（IQR） | Completeness of the videos Median（IQR） | GQS Median（IQR） | mDISCERN Median（IQR） | MQ-VET Median（IQR） |
| --- | --- | --- | --- | --- | --- |
| Video uploaders |  |  |  |  |  |
| Healthcare-related professionals/institutions | 4 (4, 4) | 30 (23, 45) | 3 (3, 5) | 3 (2, 4) | 51 (43, 62) |
| Official media/popular science institutions | 4 (4, 4) | 42 (29, 43) | 4 (4, 4) | 4 (3, 5) | 59 (50, 64) |
| General media/individual users | 4 (3, 4) | 22 (20, 38) | 3 (2, 4) | 3 (2, 3) | 42.5 (31, 53) |
| Patient/Patient family | 3 (3, 4) | 24 (22, 28) | 2 (2, 3) | 2 (1, 2) | 38 (26, 41) |
| *P* | 0.013 | 0.143 | 0.001 | <0.001 | <0.001 |
| *H* | 10.831 | 5.423 | 16.492 | 20.959 | 18.692 |
| Video present formats |  |  |  |  |  |
| Independently filmed footage | 3 (3, 4) | 22 (18, 28) | 2 (2, 3) | 2 (1, 3) | 39 (29, 43) |
| Editted videos with narration | 4 (3, 4) | 30 (24, 44.50) | 2.50 (2, 4) | 3 (2, 4) | 48 (27, 58) |
| Animation | 4 (4, 4) | 29 (27, 42) | 4 (3, 4) | 3 (3, 4) | 50 (43, 58) |
| PPT/Lecturing | 4 (4, 4) | 51 (36.50, 51) | 5 (4, 5) | 4 (3, 4) | 64 (49, 65) |
| 2 or more of the aforementioned styles | 4 (4, 4) | 40.50 (33, 46) | 4.5 (4, 5) | 3 (2, 4) | 56 (53, 59) |
| *P* | 0.008 | <0.001 | <0.001 | 0.003 | <0.001 |
| *H* | 13.82 | 22.046 | 24.419 | 15.798 | 21.524 |
| Video cotent |  |  |  |  |  |
| Technical introductions and procedural demonstrations | 3 (3, 4) | 22 (22, 28) | 2 (1, 2) | 3 (1, 3) | 28 (27, 39) |
| Popular science dissemination | 4 (3.5, 4) | 27 (20.5, 40.5) | 3 (2, 4) | 3 (2, 4) | 45 (39, 54.5) |
| Personal treatment reflections and experiential accounts | 3 (3, 3.5) | 24 (20, 27.5) | 2 (2, 2.5) | 2 (1, 2) | 37 (24, 40) |
| *P* | <0.001 | 0.339 | <0.001 | 0.001 | <0.001 |
| *H* | 16.442 | 2.163 | 16.169 | 14.655 | 19.908 |

Supplemental Table3. Video Quality Scores by different video categories on YouTube（*n*=86）

| Variables | Attitude for ECT Median（IQR） | Completeness of the videos Median（IQR） | GQS Median（IQR） | mDISCERN Median（IQR） | MQ-VET Median（IQR） |
| --- | --- | --- | --- | --- | --- |
| Video uploaders |  |  |  |  |  |
| Healthcare-related professionals/institutions | 4 (4, 4) | 35 (29.5, 39.5) | 4 (3, 4) | 3 (3, 4) | 54 (52, 59.5) |
| Official media/popular science institutions | 4 (4, 4) | 36 (33, 44) | 4 (3, 4) | 3 (3, 4) | 52 (51, 62) |
| General media/individual users | 4 (4, 4) | 26 (20, 42) | 3 (2, 4) | 3 (3, 4) | 51 (43, 59) |
| Patient/Patient family | 4 (2.5, 4) | 37 (25, 43.5) | 3 (2, 4) | 3 (2.5, 3) | 49.5 (37.5, 54.5) |
| *P* | 0.206 | 0.255 | 0.107 | 0.494 | 0.189 |
| *H* | 4.570 | 4.058 | 6.1 | 2.401 | 4.776 |
| Video present formats |  |  |  |  |  |
| Independently filmed footage | 4 (3, 4) | 26 (19, 38) | 3 (2, 4) | 3 (2, 4) | 47 (41, 54) |
| Editted videos with narration | 4 (4, 4) | 44 (35.5, 45) | 4 (3.5, 4) | 3 (3, 3.5) | 63 (60.5, 65) |
| Animation | 4 (4, 4) | 35 (24, 46) | 3 (2, 4) | 3.5 (3, 4) | 50.5 (49, 52) |
| PPT/Lecturing | 4 (4, 4) | 38 (29, 41) | 4 (3, 4) | 4 (3.5, 4.5) | 57 (53.5, 58.5) |
| 2 or more of the aforementioned styles | 4 (4, 4) | 38 (33, 43) | 4 (3, 4) | 4 (3, 4) | 56 (54, 63) |
| *P* | 0.115 | 0.009 | 0.001 | 0.031 | <0.001 |
| *H* | 7.429 | 13.579 | 18.552 | 10.622 | 24.465 |
| Video cotent |  |  |  |  |  |
| Technical introductions and procedural demonstrations | 4 (4, 4) | 37 (29, 39) | 3.5 (3, 4) | 3.5 (3, 4) | 56 (52, 57) |
| Popular science dissemination | 4 (4, 4) | 33.50 (21, 42) | 4 (2, 4) | 3 (3, 4) | 53.50 (45, 61) |
| Science report | 4 (3, 4) | 26.50 (24, 39) | 3 (3, 4) | 4 (4, 5) | 56 (49, 60) |
| Personal treatment reflections and experiential accounts | 4 (3.5, 4) | 33.50 (25, 43) | 3 (2, 4) | 3 (2.5, 3.5) | 52.5 (40.50, 54.5) |
| *P* | 0.457 | 0.898 | 0.837 | 0.080 | 0.440 |
| *H* | 2.603 | 0.595 | 0.851 | 6.772 | 2.702 |
