# Supplementary material for: Reliable, neutral, and comprehensive, or their antithesis? A cross-sectional analysis of electroconvulsive therapy-related video quality across TikTok, BiliBili, and YouTube
Source: Front Public Health. 2026 Apr 20;14:1796766. doi: 10.3389/fpubh.2026.1796766 (PMC13136114; doi:10.3389/fpubh.2026.1796766)
Supplement: Supplementary file 2 [file Table_2.docx]

1. **GQS (Global Quality Score)**

The total score is 1 to 5.

【Score 1】Poor quality, poor flow of the site, most information missing, not at all useful for patients.

【Score 2】Generally poor quality and poor flow, some information listed but many important topics missing, of very limited use to patients

【Score 3】Moderate quality, suboptimal flow, some important information is adequately discussed but others poorly discussed, somewhat useful for patients

【Score 4】Good quality and generally good flow, most of the relevant information is listed, but some topics not covered, useful for patients

【Score 5】Excellent quality and excellent flow, very useful for patients

Compared to mDISCERN, which considers video traffic, website flow, and user-friendliness, the Global Quality Score (GQS), developed by Singh et al. in 2012, is the most comprehensive, straightforward, and widely applicable assessment method for evaluating the overall quality of videos. We included two more scoring systems as a complement since GQS lacks detailed assessment and the evaluation criteria are quite arbitrary and impossible to measure.

1. **mDISCERN (Modified DISCERN)**

The score of each question ranges from 0 to 1. The total score is 0 to 5.

1. **mDISCERN-1 Are the aims clear and achieved?**

0-no 1-yes

1. **mDISCERN-2 Are reliable sources of information used?**

0-no 1-yes

1. **mDISCERN-3 Is the information presented balanced and unbiased?**

0-no 1-yes

1. **mDISCERN-4 Are additional sources of information listed for patient reference?**

0-no 1-yes

1. **mDISCERN-5 Are areas of uncertainty mentioned?**

no 1-yes

A verified rating scale and scoring criteria, the mDISCERN tool is intended to evaluate the quality of medical textual materials and associated videos. The mDISCERN tool user's guide states that movies with a total score of 3 are of intermediate quality, those with a score higher than 3 are of good quality, and those with a score lower than 3 are of bad quality. The benefits of mDISCERN include its widespread use, ease of comprehension, speed, and ease of use; but, its limited assessment dimensions and alternatives (yes or no) do not fully meet our needs for video content evaluation. For instance, not all of the questions in the mDISCERN tool ask about video traffic, which is a crucial component in determining the quality of videos.

1. **the Medical Quality Video Evaluation Tool (MQ-VET)**

**Part 1**

1. Dates of updates, if any, are clearly stated
2. The recording date of the video and date on which the information was accessed are mentioned
3. The resources and references used are clearly stated
4. Concerns about advertising and potential conflicts of interest have been resolved
5. Sufficient information was provided about the identity of the presenter in the video

**Part 2**

1. The materials used in the video facilitated learning
2. The video covered the basic concepts of the subject
3. To explain the medical topic, visual resources were used sufficiently
4. The medical terms used were well-explained

**Part 3**

10. The sound quality of the video was sufficient

11. The image quality of the video was sufficient

12. The information in the video is clear and understandable

**Part 4**

1. The video generally met my expectations
2. Information about the video content was provided at the beginning

15. The video provided new knowledge and skills

The MQ-VET is a specialized instrument designed to assess the quality of online medical video content, integrating evaluations of both production quality and informational reliability to furnish a comprehensive appraisal . This tool employs a series of detailed metrics, encompassing visual presentation, audio clarity, scientific rigor, logical coherence, and the practical utility of the content. The MQ-VET comprises 15 items, each rated on a five-point Likert scale ranging from "strongly disagree" (1) to "strongly agree" (5). Total scores span from 15 to 75, with higher aggregate scores reflecting superior overall video quality.
